# High-Power and Ultralong-Life Aqueous Zinc-Ion Hybrid Capacitors Based on Pseudocapacitive Charge Storage

**DOI:** 10.1007/s40820-019-0328-3

**Published:** 2019-10-31

**Authors:** Liubing Dong, Wang Yang, Wu Yang, Chengyin Wang, Yang Li, Chengjun Xu, Shuwei Wan, Fengrong He, Feiyu Kang, Guoxiu Wang

**Affiliations:** 1 Centre for Clean Energy Technology, Faculty of Science, University of Technology Sydney, Sydney, NSW 2007 Australia; 2 School of Chemistry and Chemical Engineering, Yangzhou University, Yangzhou, 225002 People’s Republic of China; 3 School of Photovoltaic and Renewable Energy Engineering, University of New South Wales, Sydney, NSW 2052 Australia; 4 Shenzhen Geim Graphene Center, Tsinghua Shenzhen International Graduate School, Tsinghua University, Shenzhen, 518055 People’s Republic of China; 5 HEC Group Pty Ltd, Canterbury, VIC 3216 Australia

**Keywords:** Zinc-ion hybrid capacitor, Hydrous ruthenium oxide, Ultralong life, Redox pseudocapacitance, High power

## Abstract

Rechargeable aqueous zinc-ion hybrid capacitors and zinc-ion batteries are promising safe energy storage systems. In this study, amorphous RuO_2_·H_2_O for the first time was employed to achieve fast and ultralong-life Zn^2+^ storage based on a pseudocapacitive storage mechanism. In the RuO_2_·H_2_O||Zn zinc-ion hybrid capacitors with Zn(CF_3_SO_3_)_2_ aqueous electrolyte, the RuO_2_·H_2_O cathode can reversibly store Zn^2+^ in a voltage window of 0.4–1.6 V (vs. Zn/Zn^2+^), delivering a high discharge capacity of 122 mAh g^−1^. In particular, the zinc-ion hybrid capacitors can be rapidly charged/discharged within 36 s with a very high power density of 16.74 kW kg^−1^ and a high energy density of 82 Wh kg^−1^. Besides, the zinc-ion hybrid capacitors demonstrate an ultralong cycle life (over 10,000 charge/discharge cycles). The kinetic analysis elucidates that the ultrafast Zn^2+^ storage in the RuO_2_·H_2_O cathode originates from redox pseudocapacitive reactions. This work could greatly facilitate the development of high-power and safe electrochemical energy storage.

**Electronic supplementary material:**

The online version of this article (10.1007/s40820-019-0328-3) contains supplementary material, which is available to authorized users.

## Highlights

- This work starts the research of pseudocapacitive oxide materials for multivalent Zn^2+^ storage.
- The constructed RuO_2_·H_2_O||Zn systems exhibit outstanding electrochemical performance, including a high discharge capacity, ultrafast charge/discharge capability, and excellent cycling stability.
- The redox pseudocapacitive behavior of RuO_2_·H_2_O for Zn^2+^ storage is revealed.

**Electronic supplementary material:**

The online version of this article (10.1007/s40820-019-0328-3) contains supplementary material, which is available to authorized users.

## Graphical abstract

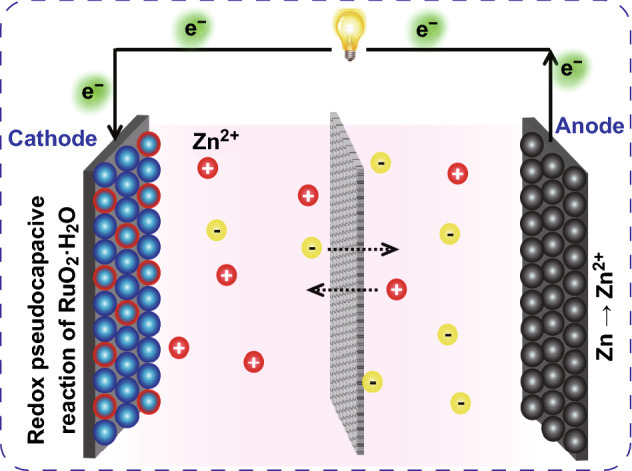

## Introduction

Novel energy storage systems with the merits of high safety, fast charge–discharge capability, and high energy density are highly demanded with the rapid development of electric vehicles and customer electronics. Recently, multivalent-ion (e.g., Zn^2+^, Ca^2+^, Mg^2+^, and Al^3+^) storage systems have emerged and exhibited unique electrochemical behaviors [1–6]. During various multivalent-ion storage systems, zinc metal anode-based aqueous rechargeable zinc-ion batteries (ZIBs) and zinc-ion hybrid capacitors (ZICs) are particularly attractive [1, 7–11], due to their high safety, low cost, abundant natural resource of zinc, and unique electrochemical features of zinc metal anodes such as low redox potential of − 0.76 V (vs. standard hydrogen electrode) and ultrahigh volumetric capacity of 5845 Ah L^−1^. Furthermore, the high ionic conductivity of aqueous electrolytes such as ZnSO_4_ solutions in ZIBs and ZICs is beneficial for achieving high power output. The electrochemical properties of ZIBs and ZICs are strongly dependent on the Zn^2+^-storage behaviors in cathode materials.

Several cathode materials have been developed for ZIBs and ZICs, including manganese oxides, vanadium oxides, Prussian blue analogs, conductive polymers, and carbon materials. Zn^2+^ insertion/extraction in manganese oxides, especially tunnel-structured MnO_2_, creates high specific capacities. However, the poor electrical conductivity of manganese oxides and manganese dissolution issues cause unsatisfactory rate performance and poor cycling stability [12–15]. Vanadium oxides possess high capacities and fast kinetics for Zn^2+^ storage [16–22], whereas their high toxicity impedes their practical applications. Besides, most of the Prussian blue analogs show low capacities of about 50 mAh g^−1^ when used as cathode materials for ZIBs [23–26]. Although conductive polymers (e.g., polyaniline and polypyrrole) and carbon materials (e.g., activated carbon, denoted as “AC”) generally have a Zn^2+^-storage capacity of 100–150 mAh g^−1^ and better rate performance compared to manganese oxides [11, 27–30], their low density of about 0.3–1 mg cm^−2^ is unfavorable for the volumetric energy density of corresponding batteries. Therefore, seeking high-performance Zn^2+^-storage materials is still a big challenge.

Herein, for the first time, we demonstrate that fast, ultralong-life, and safe Zn^2+^ storage can be realized in amorphous RuO_2_·H_2_O cathode materials based on a pseudocapacitive storage mechanism. The constructed RuO_2_·H_2_O||Zn ZICs can reversibly store Zn^2+^ in a voltage window of 0.4–1.6 V (vs. Zn/Zn^2+^), delivering a capacity of about 122 mAh g^−1^, an excellent rate capability and an ultralong cycle life exceeding 10,000 cycles.

## Experimental

### Electrochemical Measurements

Amorphous ruthenium oxide hydrate (RuO_2_·*x*H_2_O) powder was obtained from Sigma-Aldrich Corporation. To synthesize anhydrous RuO_2_, the RuO_2_·*x*H_2_O powder was heat-treated in air at 300 °C for 1 h with a heating rate of 5 °C min^−1^. The amorphous RuO_2_·*x*H_2_O power (or anhydrous RuO_2_ powder) was mixed with conductive black and polyvinylidene fluoride binder in *N*-methyl-pyrrolidone solutions, then coated on a stainless steel foil, and finally dried at 100 °C in vacuum to obtain RuO_2_·*x*H_2_O (or RuO_2_) electrodes. Mass loading of active materials in the prepared cathodes was 2.5–3.0 mg cm^−2^. Electrochemical performance of these ruthenium oxides for Zn^2+^ storage was evaluated by assembling CR2032 coin cells, in which RuO_2_·*x*H_2_O (or RuO_2_) electrode was used as the cathode, commercial Zn foil was used as the anode, air-laid paper was used as separator, and 2 M Zn(CF_3_SO_3_)_2_ or 2 M ZnSO_4_ aqueous solution served as the electrolyte. Cyclic voltammetry (CV) and electrochemical impedance spectroscopy (EIS) tests were performed on a Bio-Logic VMP3 electrochemical station. An AC amplitude of 5 mV and a frequency range of 0.1–100 kHz were applied for the EIS test at open-circuit voltage (OCV). For galvanostatic charge–discharge (GCD) measurements, when the applied current was 0.1–3 A g^−1^, they were performed on a LAND battery testing instrument, and when the current was 5–20 A g^−1^, the GCD measurements were completed on the Bio-Logic VMP3 electrochemical station. (This is because for fast charge/discharge tests, Bio-Logic VMP3 electrochemical station is more sensitive and accurate.)

### Material and Electrode Characterizations

We used scanning electron microscopy (SEM; model: Zeiss Supra 55VP) and transmission electron microscopy (TEM; model: Tecnai G2 F30) to observe the micromorphologies of samples and used a Brunauer–Emmett–Teller (BET) analyzer to characterize the specific surface area. X-ray diffraction (XRD; model: Bruker D8 Discover Diffractometer) and X-ray photoelectron spectroscopy (XPS; model: MDTC-EQ-M20-01) were applied to analyze the phase and compositions. Thermogravimetric (TG)-differential scanning calorimeter was utilized to determine the water content in amorphous RuO_2_·*x*H_2_O powder. Note that to characterize the electrodes at various charge/discharge states, corresponding cells were charged/discharged, then disassembled, and washed using deionized water five times to remove surface-adsorbed electrolyte.

## Results and Discussion

**Fig. 1:**
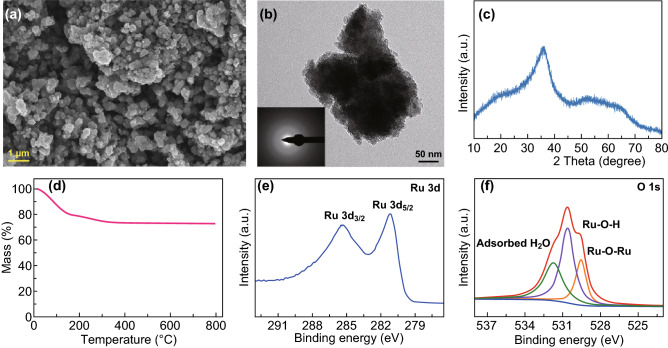
**a** SEM image, **b** TEM image (inset: SAED pattern), **c** XRD pattern, **d** TG curve, **e** Ru 3d, and **f** O1 s XPS spectra of the RuO_2_·*x*H_2_O sample

Figure 1 shows the physicochemical characteristics of the RuO_2_·*x*H_2_O sample. The RuO_2_·*x*H_2_O is irregular-shaped particles with a size of about 100–500 nm (Fig. 1a). Its selected-area electron diffraction (SAED) image (inset in Fig. 1b) exhibits a characteristic halo ring pattern, revealing the amorphous feature of the RuO_2_·*x*H_2_O. Correspondingly, the high-resolution TEM image does not show clear lattice fringes (Fig. S1). The amorphous feature of the RuO_2_·*x*H_2_O is also confirmed by the XRD result (Fig. 1c), from which only several broad diffraction peaks are observed. To determine the structural water content in the RuO_2_·*x*H_2_O, TG analysis was performed, as shown in Fig. 1d. Mass loss in the temperature range of 100–300 °C originates from the structural water of the RuO_2_·*x*H_2_O [31], which is ~ 12.4 wt%. This means that *x* in the RuO_2_·*x*H_2_O is 1.0. Therefore, the RuO_2_·*x*H_2_O is denoted as RuO_2_·H_2_O. In the XPS spectrum of Ru 3d (Fig. 1e), the Ru 3d_3/2_ peak at 285.3 eV and Ru 3d_5/2_ peak at 281.1 eV are observed, corresponding well to the previously reported hydrous RuO_2_ [32, 33]. Ru 3p XPS spectrum shown in Fig. S2 provides consistent evidence. O 1 s XPS spectrum shown in Fig. 1f is split into three peaks, proving the coexistence of Ru–O–Ru and Ru–O–H bonds in the amorphous RuO_2_·H_2_O [32, 33].

**Fig. 2:**
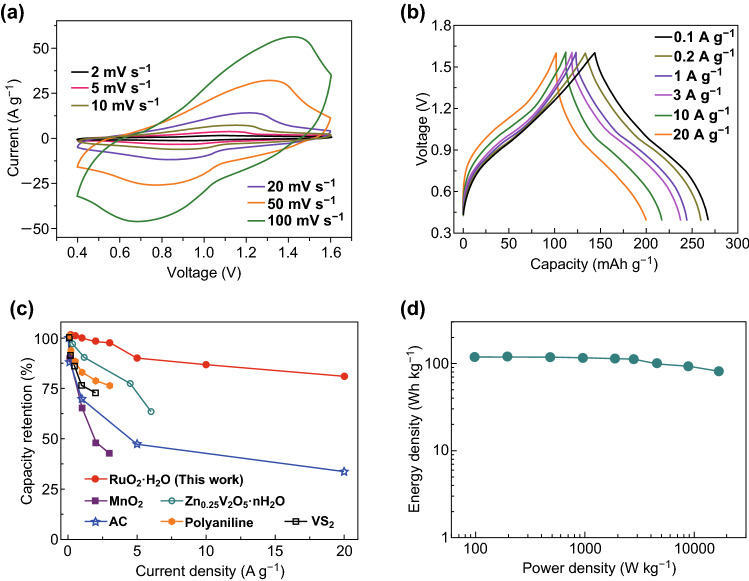
Electrochemical properties of the RuO_2_·H_2_O||Zn systems: **a** CV curves, **b** GCD profiles, **c** rate performance (in comparison with previously reported cathode materials for Zn^2+^ storage), and **d** Ragone plot

The electrochemical performance of the RuO_2_·H_2_O for Zn^2+^ storage was evaluated by assembling the cells with RuO_2_·H_2_O cathode, Zn metal anode, and Zn(CF_3_SO_3_)_2_ aqueous electrolyte. The RuO_2_·H_2_O||Zn system shows an open-circuit voltage of 1.05 V and can be reversibly charged/discharged in a voltage window of 0.4–1.6 V (Fig. 2a). In such a voltage window, the Zn(CF_3_SO_3_)_2_ and ZnSO_4_ aqueous electrolytes are stable and water decomposition does not occur (Fig. S3). In the CV curves, there is one pair of broad redox peaks. Even at high scan rates such as 100 mV s^−1^, the redox peaks remain, suggesting good rate performance of the RuO_2_·H_2_O||Zn system. As shown in the GCD profiles (Fig. 2b), the charge curves and discharge curves deviate from linear shapes without flat voltage plateaus. This is consistent with the broad redox peaks observed in the CV curves. At a charge/discharge current of 0.1 A g^1^, the RuO_2_·H_2_O cathode shows a discharge capacity of 122 mAh g^−1^ with a coulombic efficiency of 86%. When the current increases for 200 times (to 20 A g^−1^), in which the RuO_2_·H_2_O||Zn system is charged/discharged within 36 s, the discharge capacity still reaches 98 mAh g^−1^. In fact, considering that the coulombic efficiency of the RuO_2_·H_2_O||Zn system at low current densities of 0.1–1 A g^−1^ and high current densities of 3–20 A g^−1^ is 86–98% and 99–100%, respectively, the RuO_2_·H_2_O||Zn system is more suitable for fast charging/discharging. For comparison, rate performance of some typical cathode materials for Zn^2+^ storage is summarized in Fig. 2c, including MnO_2_ [15], Zn_0.25_V_2_O_5_·*n*H_2_O [16], VS_2_ [34], polyaniline [28], and AC [11]. Figure 2c intuitively shows the excellent rate capability of the RuO_2_·H_2_O cathode, compared with the other cathode materials. It should be noted that the RuO_2_·H_2_O exhibits similar superior performance in 2 M ZnSO_4_ aqueous electrolyte (Fig. S4). Furthermore, according to the Ragone plot shown in Fig. 2d, the RuO_2_·H_2_O cathode can provide a maximum energy density of 119 Wh kg^−1^. More importantly, it keeps a high energy density of 82 Wh kg^−1^ under the condition of delivering an ultrahigh power output of 16.74 kW kg^−1^. Such a high power output with considerable energy density is almost impossible for most of the current electrochemical energy storage systems [9, 16, 35]. For instance, the maximum power density of currently reported lithium-ion batteries and aqueous ZIBs is generally smaller than 1–10 kW kg^−1^.

**Fig. 3:**
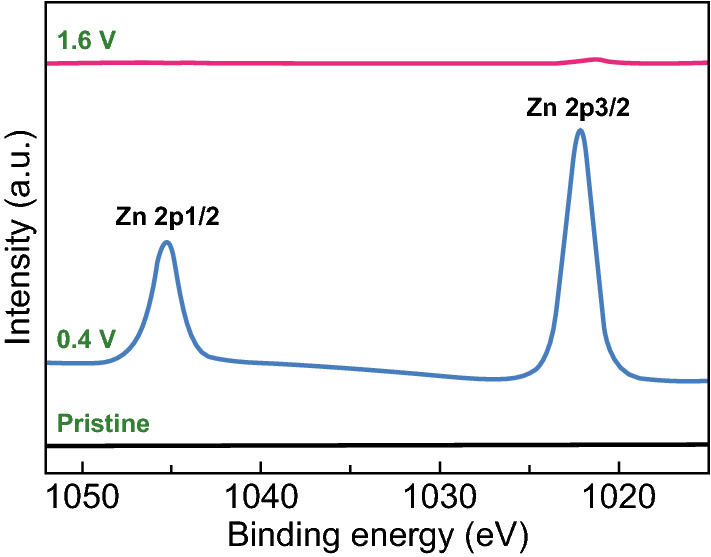
High-resolution Zn 2p XPS spectra of the RuO_2_·H_2_O cathode at various states

**Fig. 4:**
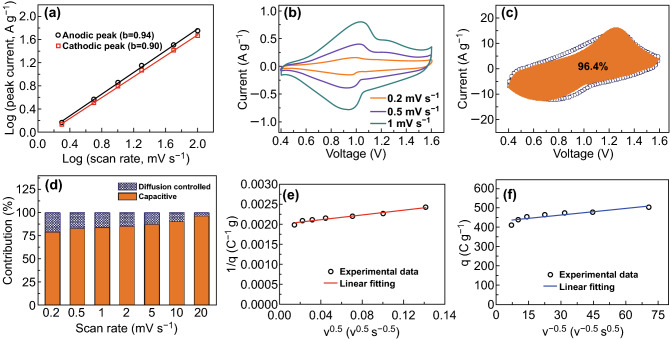
Kinetic analysis of Zn^2+^ storage in the RuO_2_·H_2_O: **a** relationship curve of peak current versus scan rate, **b** CV curves at low scan rates of 0.2–1 mV s^−1^, **c** capacitive contribution (orange region) to the total current at 20 mV s^−1^, **d** summary of the contribution ratios of capacitive capacity and diffusion-controlled capacity, **e, f** capacitive contribution analyzed through Trasatti’s method, in which *q* and *v* are charge stored and scan rate, respectively

The kinetic analysis was performed to reveal the mechanisms for the superior electrochemical performance of the RuO_2_·H_2_O cathode. Zn^2+^ storage in the RuO_2_·H_2_O cathode was firstly confirmed by the high-resolution Zn 2p XPS spectra shown in Fig. 3. Zn^2+^ ions were stored in the RuO_2_·H_2_O cathode when the cathode was discharged from pristine state to 0.4 V, and almost all Zn^2+^ ions were extracted from the RuO_2_·H_2_O cathode when the cathode was further charged to 1.6 V, implying highly reversible Zn^2+^ storage in the RuO_2_·H_2_O cathode. Besides, H^+^ from the slightly acid Zn(CF_3_SO_3_)_2_ aqueous electrolyte is also proved to participate in the electrochemical reactions in the RuO_2_·H_2_O//Zn system and contributes to a small capacity to the RuO_2_·H_2_O cathode (Figs. S5–S7). For the CV curves at various scan rates of the RuO_2_·H_2_O||Zn system (Fig. 2a), the relationship between their peak current (*i*) and scan rate (*v*) can be depicted through Eq. 1 [36]:

1 $$i = av^{b} ,$$

where *a* and *b* are variable parameters. Particularly, *b* values of 0.5 and 1.0 represent a diffusion-controlled process and a complete capacitive process, respectively [33]. As shown in Fig. 4a, the *b* values for the anodic peaks and cathodic peaks are close to 1.0, suggesting that Zn^2+^ storage in the RuO_2_·H_2_O cathode is dominated by a capacitive process.

We further tested CV curves at low scan rates (Fig. 4b). At 0.2–1 mV s^−1^, the voltage separation between anodic peaks and cathodic peaks is very small (< 0.08 V), which is a typical feature of pseudocapacitive behavior [37]. As a comparison, ZIB cathode materials such as MnO_2_ generally possess a large voltage separation (> 0.3 V; Fig. S8). Furthermore, two capacitance differentiation methods were applied to analyze the pseudocapacitive reaction of the RuO_2_·H_2_O for Zn^2+^ storage. According to Dunn’s method (Fig. 4c, d) [37], 79.0–96.4% capacitance originates from the surface-controlled capacitive process, i.e., redox pseudocapacitance and electric double-layer capacitance. Considering that the specific surface area of the RuO_2_·H_2_O is only 57 m^2^ g^−1^ (Fig. S9), the majority of the capacitance is redox pseudocapacitance, while the electric double-layer capacitance accounts a small fraction. Trasatti’s method analysis in Fig. 4e, f points out that the maximum charge that can be stored in the RuO_2_·H_2_O and the charge stored at the so-called outer surface (easily accessible to electrolyte ions) of the RuO_2_·H_2_O are 502.5 and 428.4 C g^−1^, respectively [38]. This means that 85.3% capacity is from the outer surface, which is consistent with the Dunn’s method analysis. Such an energy storage mechanism of redox pseudocapacitive behavior, as well as high conductivity of hydrous ruthenium oxides (higher than 100 S cm^−1^) [31], benefits for the ultrafast charging/discharging of the RuO_2_·H_2_O cathode [37].

**Fig. 5:**
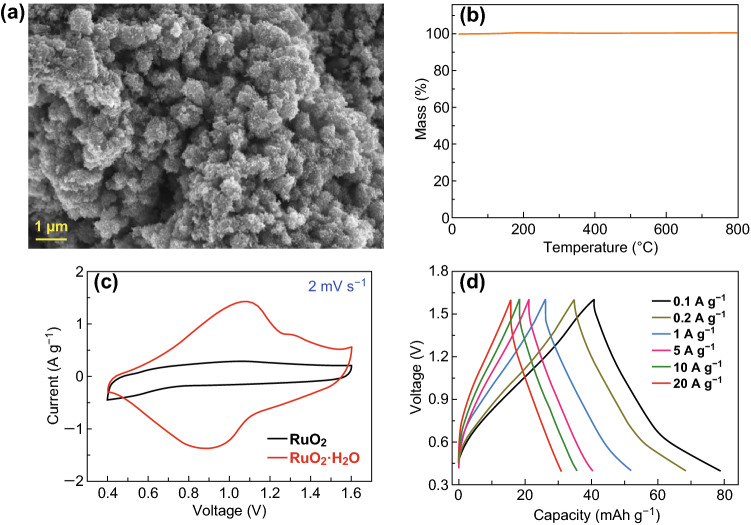
**a** SEM image and **b** TG curve of the anhydrous RuO_2_ sample. Electrochemical behaviors of the anhydrous RuO_2_||Zn system with 2 M Zn(CF_3_SO_3_)_2_ aqueous electrolyte: **c** CV curve (in comparison with that of RuO_2_·H_2_O||Zn system) and **d** GCD profiles at 0.1–20 A g^−1^

It should be emphasized that the structural water in the RuO_2_·H_2_O plays a vital role in Zn^2+^ storage. As a comparison, anhydrous RuO_2_ sample was synthesized by heat-treating the RuO_2_·H_2_O in air (Figs. 5a, b and S10). The TG curve confirms that after heat treatment, structural water content of the sample is negligible. As for the anhydrous RuO_2_, its redox pseudocapacitive reactions are notably suppressed, corresponding to very low Zn^2+^-storage capacities of 38–15 mAh g^−1^ at 0.1–20 A g^−1^ (Figs. 5c, d and S11, S12), even though anhydrous RuO_2_ generally possesses a higher electrical conductivity than hydrous RuO_2_·*x*H_2_O [31]. This is because the structural water can facilitate rapid ion transport in the RuO_2_·H_2_O [34]. Similarly, hydrous ruthenium oxides perform much better than anhydrous RuO_2_ in supercapacitors with H_2_SO_4_ aqueous electrolytes [31].

**Fig. 6:**
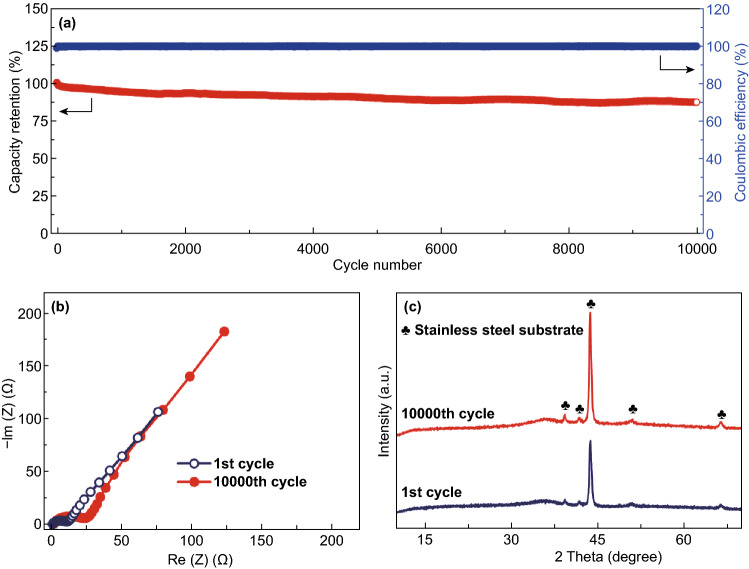
**a** Cycling stability test at 20 A g^−1^ of the RuO_2_·H_2_O||Zn system. **b** Nyquist plots and **c** XRD patterns of the RuO_2_·H_2_O cathode before and after the cycling test

Besides the excellent high rate performance, the amorphous RuO_2_·H_2_O cathode also exhibits superior long-term cyclic stability, with an 87.5% capacity retention over 10,000 charge/discharge cycles (Fig. 6a). Meanwhile, the coulombic efficiency always maintains ~ 100% during the cycling test (except for the initial tens of cycles). Nyquist plots in Fig. 6b of the RuO_2_·H_2_O||Zn hybrid capacitor reveal a small charge-transfer resistance even after the 10,000 charge/discharge cycles. In addition, the long-term cycling test does not cause an obvious change in the phase composition and micromorphology of the amorphous RuO_2_·H_2_O cathode (Fig. 6c and S13). These imply the high electrochemical and structural stability of the RuO_2_·H_2_O cathode during repeated Zn^2+^ storage processes.

## Conclusions

In summary, amorphous RuO_2_·H_2_O was employed to achieve fast, ultralong-life, and safe Zn^2+^ storage. In the RuO_2_·H_2_O||Zn zinc-ion hybrid capacitors with aqueous Zn(CF_3_SO_3_)_2_ electrolyte, the RuO_2_·H_2_O cathode reversibly stores Zn^2+^ in a voltage window of 0.4–1.6 V (vs. Zn/Zn^2+^), displaying a discharge capacity of 122 mAh g^**−**1^ and an outstanding high rate performance. The zinc-ion hybrid capacitors can be rapidly charged/discharged within 36 s, in which case a very high power density of 16.74 kW kg^**−**1^ and a high energy density of 82 Wh kg^**−1**^ are delivered. Such an excellent high rate performance originates from redox pseudocapacitive reactions of the RuO_2_·H_2_O by storing Zn^2+^. Besides, the zinc-ion hybrid capacitors exhibit superior cycling stability with 87.5% capacity retention over 10,000 charge/discharge cycles. This work could greatly facilitate the development of ultrafast and safe aqueous electrolyte-based electrochemical energy storage.

## Electronic supplementary material

Below is the link to the electronic supplementary material.

Supplementary material 1 (PDF 1627 kb)
