# Supplementary material for: High-Power and Ultralong-Life Aqueous Zinc-Ion Hybrid Capacitors Based on Pseudocapacitive Charge Storage
Source: Nanomicro Lett. 2019 Oct 31;11:94. doi: 10.1007/s40820-019-0328-3 (PMC7770721; doi:10.1007/s40820-019-0328-3)
Supplement: Supplementary file 1 — Supplementary material 1 (PDF 1627 kb) [file 40820_2019_328_MOESM1_ESM.pdf]

Supporting Information for

High-Power and Ultralong-Life Aqueous Zinc-Ion Hybrid Capacitors Based on Pseudocapacitive Charge Storage

Liubing Dong [1, †], Wang Yang [1, †], Wu Yang [1], Chengyin Wang [2], Yang Li [3], Chengjun Xu [4, *], Shuwei Wan [5], Fengrong He [5], Feiyu Kang [4], Guoxiu Wang [1, *]

[1]Centre for Clean Energy Technology, Faculty of Science, University of Technology Sydney, NSW 2007, Australia

[2]School of Chemistry and Chemical Engineering, Yangzhou University, Yangzhou 225002, People's Republic of China

[3]School of Photovoltaic and Renewable Energy Engineering, University of New South Wales, NSW 2052, Australia

[4]Shenzhen Geim Graphene Center, Tsinghua Shenzhen International Graduate School, Tsinghua University, Shenzhen 518055, People's Republic of China

[5]HEC Group Pty Ltd, VIC 3216, Australia

[†] These authors contributed equally to this work

*Corresponding authors. E-mail: vivaxuchengjun@163.com (C. Xu); guoxiu.wang@uts.edu.au (G. Wang)

Supplementary Figures and Disscussion

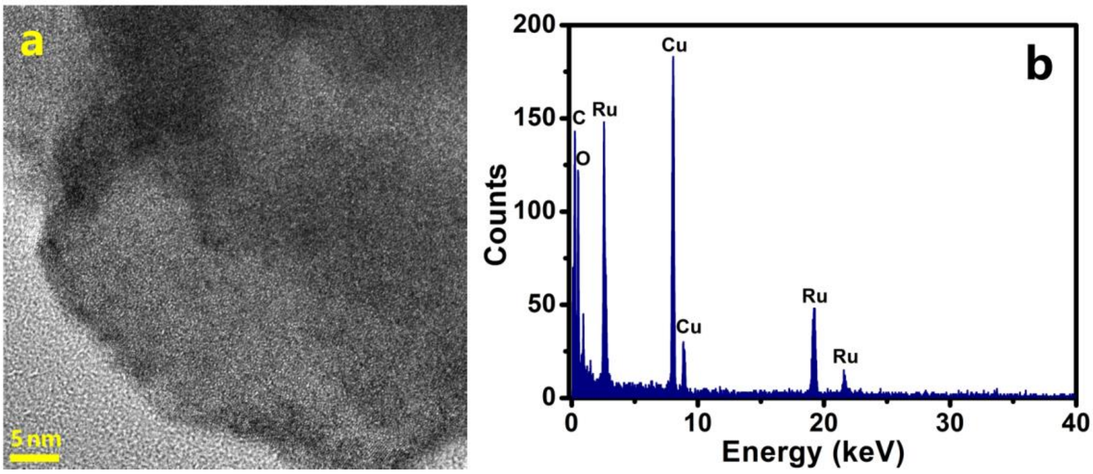

Fig. S1 a High-resolution TEM image and **b** corresponding energy-dispersive X-ray spectroscopy (EDS) analysis of the $RuO_2 \cdot xH_2O$

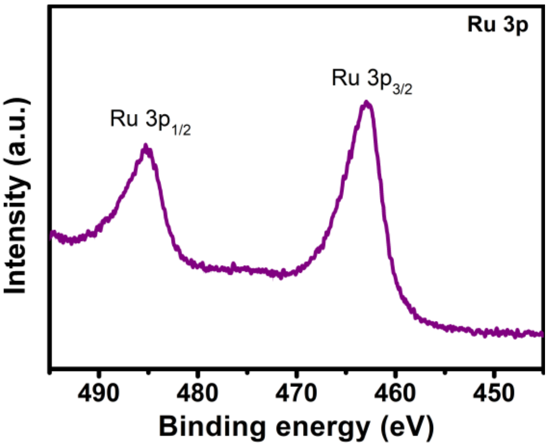

Fig. S2. High-resolution Ru 3p XPS spectrum of the $RuO_2 \cdot xH_2O$

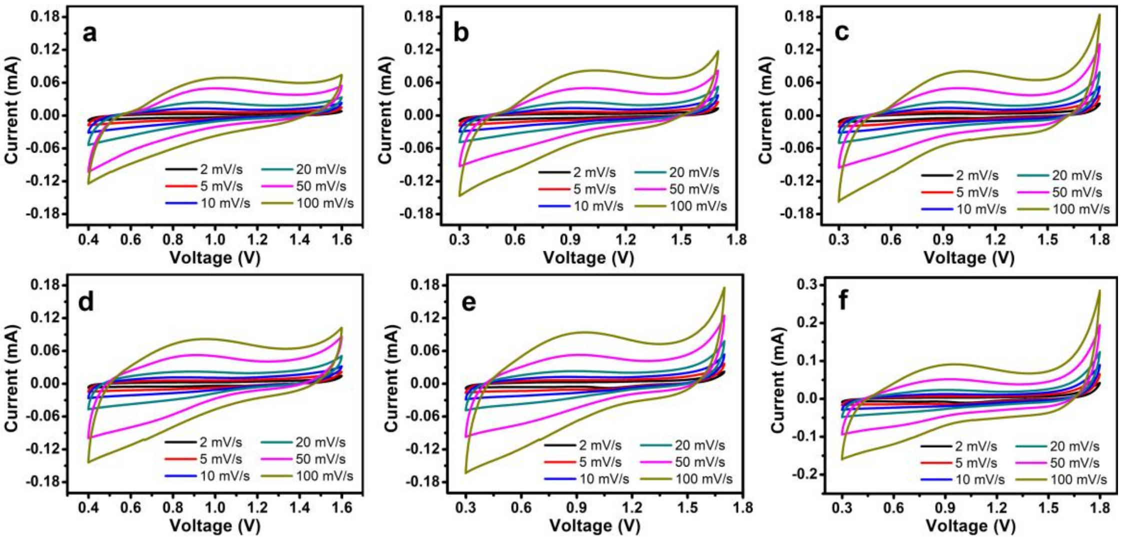

Fig. S3 CV curves of the **a-c** $Zn(CF_3SO_3)_2$ and **d-f** $ZnSO_4$ aqueous electrolytes in different voltage windows: **a, d** 0.4-1.6 V; **b, e** 0.3-1.7 V, and **c, f** 0.3-1.8 V. The CV curves were recorded in 2 M $Zn(CF_3SO_3)_2$ or 2 M $ZnSO_4$ aqueous electrolyte, and meanwhile, zinc foil was used as both reference electrode and counter electrode, and stainless steel foil was used as working electrode. We can see that in the voltage window of 0.4-1.6 V, both $Zn(CF_3SO_3)_2$ and $ZnSO_4$ aqueous electrolytes are stable at different scan rates.

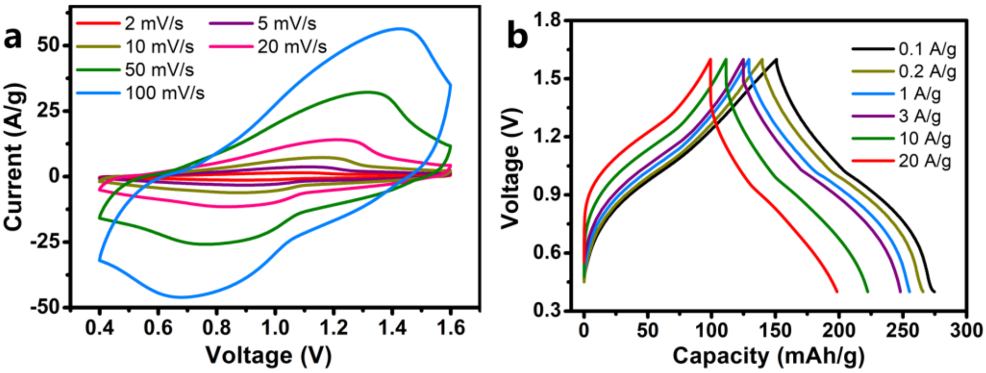

Fig. S4 a CV curves at 2-100 mV/s and **b** GCD profiles at 0.1-20 A/g of the $RuO_2 \cdot H_2O$||Zn system with 2 M $ZnSO_4$ aqueous electrolyte

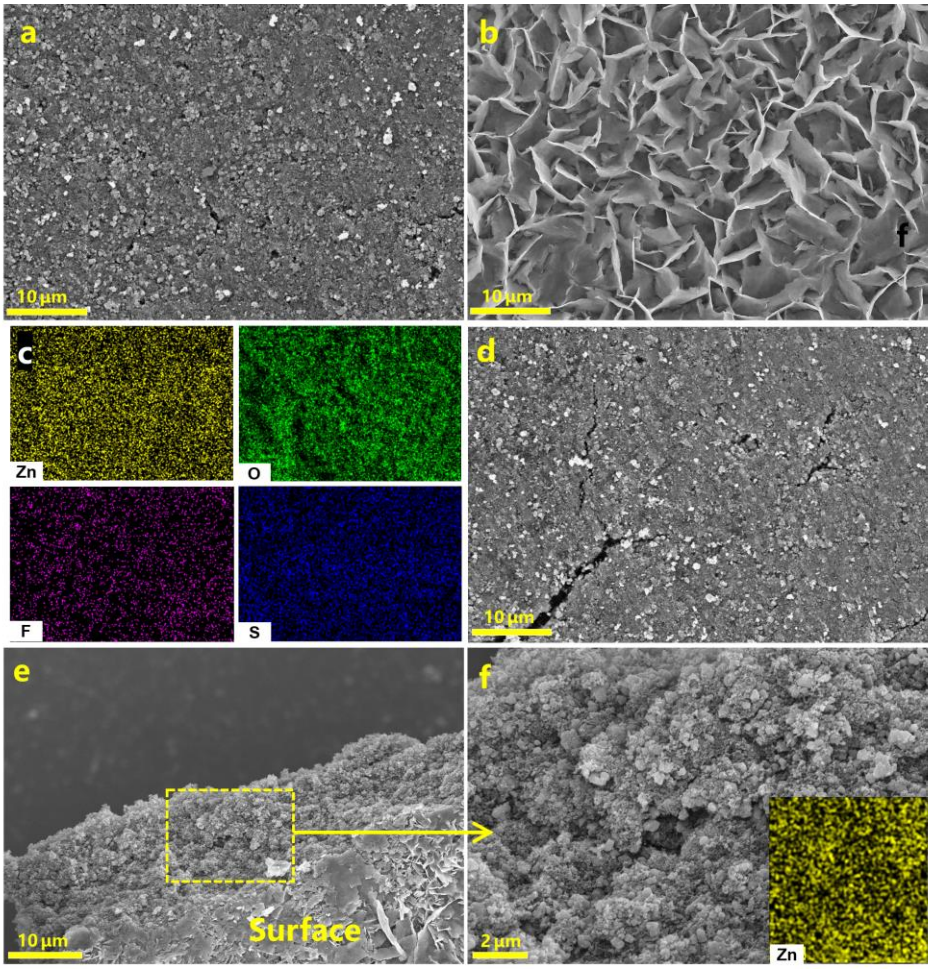

Fig. S5 Surface morphology and EDS mapping of the $RuO_2 \cdot H_2O$ cathode at various charge/discharge states in 2 M $Zn(CF_3SO_3)_2$ aqueous electrolyte: **a** original state, **b, c** fully discharged state (to 0.4 V) and **d** fully charged state (to 1.6 V). **e, f** Cross-sectional SEM images of the fully discharged $RuO_2 \cdot H_2O$ cathode. Inset in **f** shows the distribution of zinc element inside the cathode

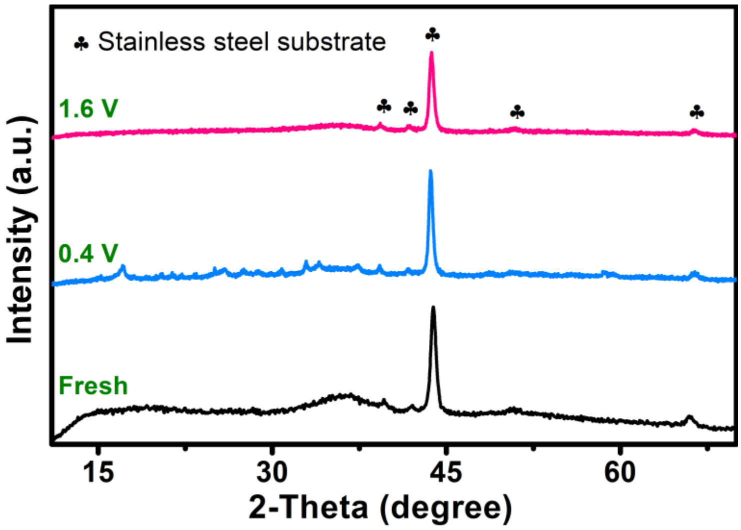

Fig. S6 XRD patterns of the $RuO_2 \cdot H_2O$ cathode at various charge/discharge states in 2 M $Zn(CF_3SO_3)_2$ aqueous electrolyte

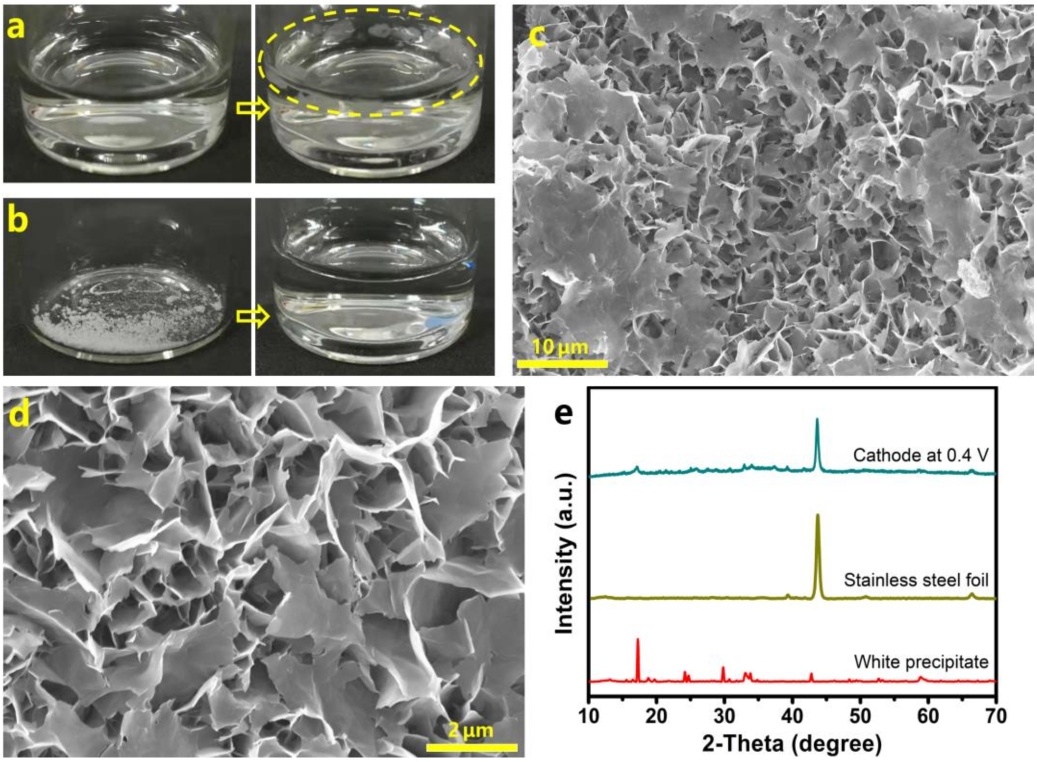

Fig. S7 Photographs showing **a** the formation of an insoluble product (we call it "white precipitate" in the following) when adding KOH solution into $Zn(CF_3SO_3)_2$ aqueous solution (molar ratio of KOH to $Zn(CF_3SO_3)_2$ is 1: 100) and **b** the dissolution of the while precipitate powder in HCl solution. **c, d** SEM images and **e** XRD pattern of the white precipitate. As a comparison, the XRD pattern of the $RuO_2{\cdot}H_2O$ cathode at 0.4 V in 2 M $Zn(CF_3SO_3)_2$ aqueous electrolyte is also presented in **e**

Discussion about Figures S5-S7

From Fig. S5, we can see that when the $RuO_2{\cdot}H_2O$ cathode is first discharged to 0.4 V, many nanosheets containing Zn, O, F, and S elements appear on the cathode surface (note that these nanosheets do not exist inside the cathode). After further charging to 1.6 V, the cathode recovers to its original morphology, accompanying with the disappear of these nanosheets. According to XRD analysis in Fig. S6, the fresh cathode and the fully charged cathode (*i.e.*, at 1.6 V) have the same phase composition, *i.e.*, amorphous $RuO_2{\cdot}H_2O$, while a new phase with the characteristic diffraction peaks at $2\theta=17.1^{o}$ and so on appears on the fully discharged cathode (*i.e.*, at 0.4 V). Apparently, the new phase detected at the fully discharged state corresponds to the nanosheets in Fig. S5b. Further, the nanosheets in Fig. S5b, c contain F and S elements, and the $Zn(CF_3SO_3)_2$ electrolyte is the only source of these two elements, therefore the nanosheets are considered to precipitate from the electrolyte. Inspired by this, we designed some experiments to study the nanosheets (Fig. S7). We added OH^- into $Zn(CF_3SO_3)_2$ aqueous solution and found that white precipitate formed (Fig. S7a). SEM observation and XRD analysis in Fig. S7c-e confirm that the white precipitate is the nanosheets in Fig. S5b. The nanosheets are soluble in acid solutions (Fig. S7b). In a word, the nanosheets irreversibly form/disappear with increased/decreased pH of the $Zn(CF_3SO_3)_2$ aqueous electrolyte. Very similar phenomenon have been reported in aqueous $Zn||ZnSO_4||MnO_2$ batteries: when the electrolyte pH increases, basic zinc sulfate nanosheets with chemical formula of $Zn_4SO_4(OH)_6{\cdot}xH_2O$ form, and when the electrolyte pH decreases, the $Zn_4SO_4(OH)_6{\cdot}xH_2O$ nanosheets dissolve into $ZnSO_4$ electrolyte [S1-S4]. According to the

above discussion, the nanosheets in Fig. S5b can be written as $Zn(CF_3SO_3)_2[Zn(OH)_2]_3\cdot xH_2O$ [S1] and their formation at fully discharged state implies an enhanced pH value of the electrolyte, which is caused by the intercalation of H^+ from the slightly acid $Zn(CF_3SO_3)_2$ electrolyte into the $RuO_2\cdot H_2O$, while the dissolution of the nanosheets during charging process (Fig. S5d) means that H^+ can reversibly extract from the $RuO_2\cdot H_2O$, leading to a decreased pH value of the electrolyte.[S1-S4] We would like to emphasize that H^+ storage only contributes to a small capacity to the $RuO_2\cdot H_2O$ cathode, due to very low H^+ concentration (~10^{-4} *vs.* 2 mol L^{-1} for Zn^{2+} concentration) of the 2 M $Zn(CF_3SO_3)_2$ electrolyte. This indirectly suggests that the high discharge capacity of the $RuO_2\cdot H_2O$ cathode originates from Zn^{2+} storage, which is also confirmed by XPS analysis in Fig. 3 and SEM-EDS mapping in Fig. S5f.

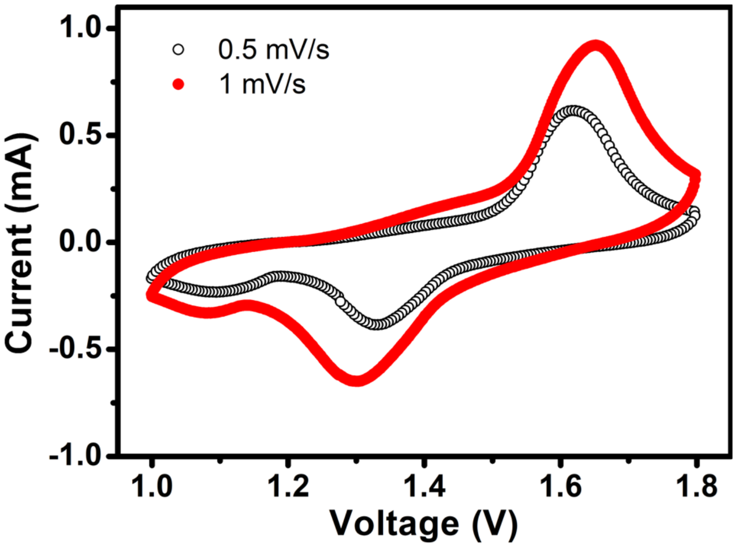

Fig. S8 Typical CV curves at 0.5-1 mV/s of MnO_2||Zn ZIBs with 2 M $Zn(CF_3SO_3)_2$ aqueous electrolytes. The voltage separation between anodic peak and cathodic peak exceeds 0.30 V at 0.5 mV/s and 0.35 V at 1 mV/s

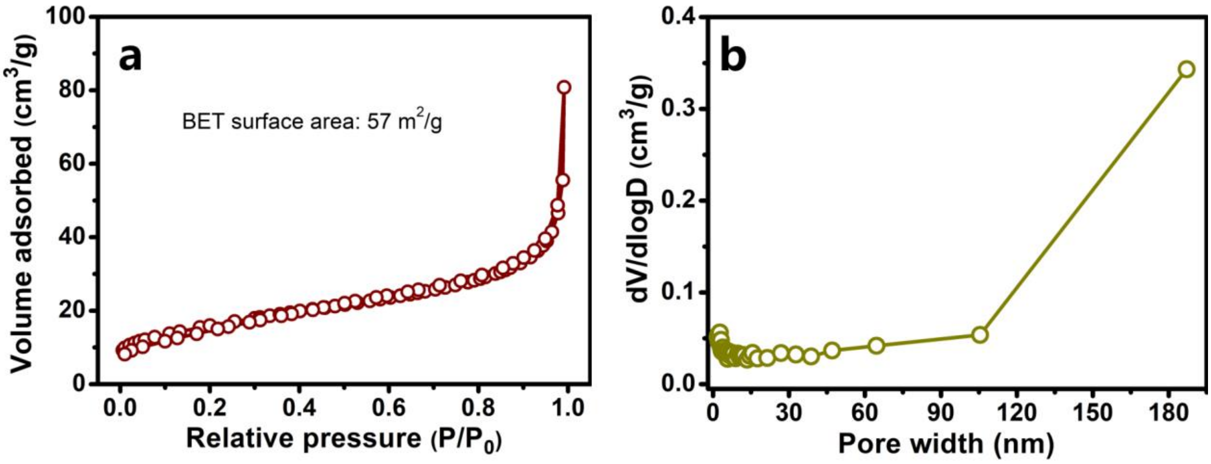

Fig. S9 a N_2 adsorption-desorption isotherm and **b** pore size distribution curve of the $RuO_2\cdot H_2O$ sample

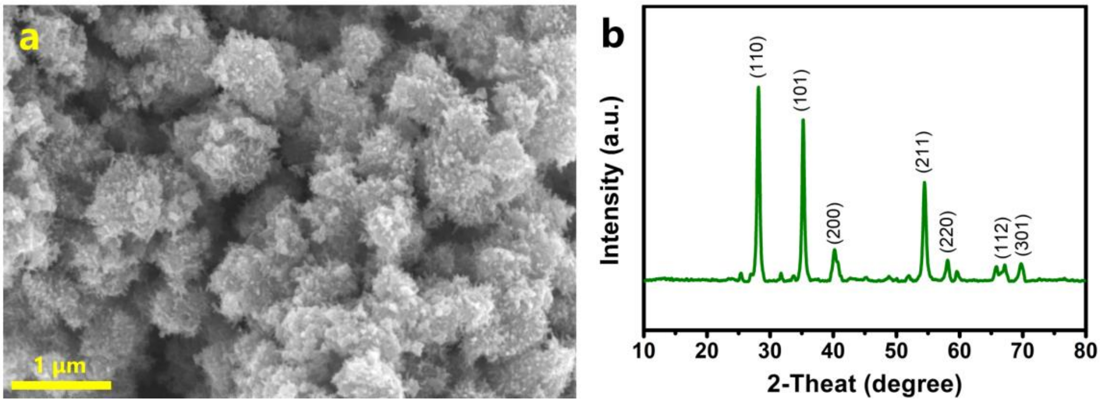

Fig. S10 **a** SEM image and **b** XRD pattern of the anhydrous RuO_2 sample. The anhydrous RuO_2 was prepared by heat-treating the $RuO_2 \cdot H_2O$ sample in air at 300 °C for 1 h. In the XRD pattern, sharp diffraction peaks can be observed, which correspond to the crystal planes of (110), (101) and so on of RuO_2 (PDF#21-1172)

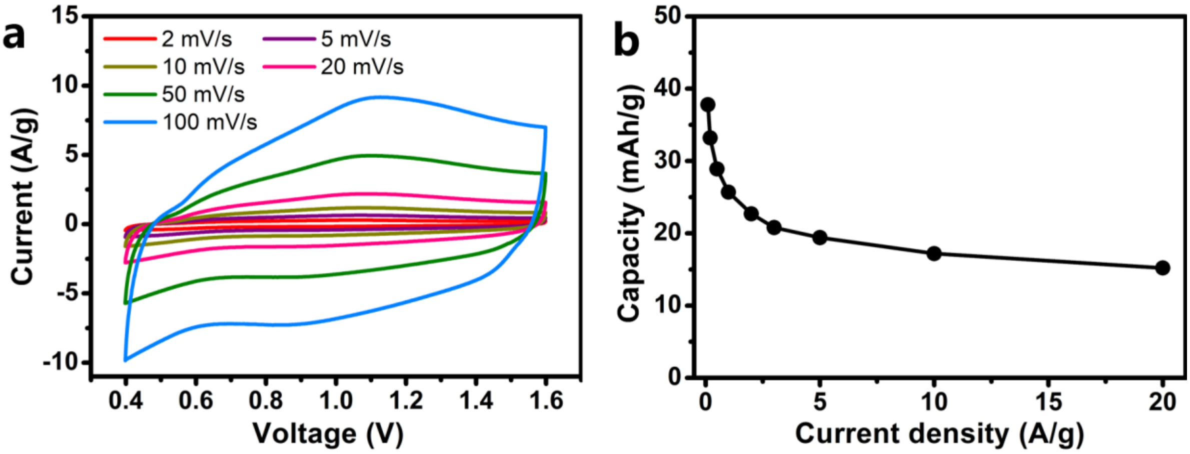

Fig. S11 Electrochemical behaviors of the anhydrous RuO_2||Zn system with 2 M $Zn(CF_3SO_3)_2$ aqueous electrolyte: **a** CV curves at 2-100 mV/s and **b** the relationship curve of discharge capacity summary *vs.* current density. OCV of the anhydrous RuO_2||Zn system is 0.73 V, low than that of the $RuO_2 \cdot H_2O$||Zn system (1.05 V). As a consequence, the anhydrous RuO_2 shows a low Zn^{2+}-storage capacity of 38 mAh/g and unsatisfactory rate performance.

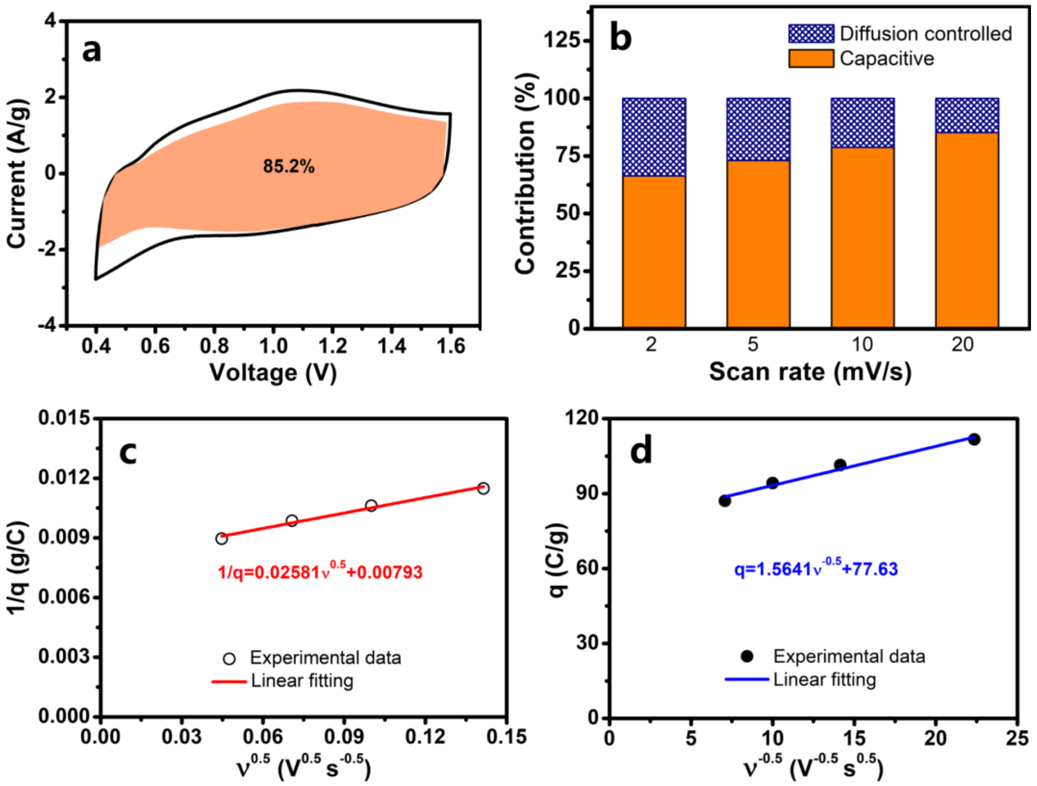

Fig. S12 Kinetic analysis of Zn^{2+} storage in the anhydrous RuO_2: **a** capacitive contribution (orange region) to the total current at 20 mV/s; **b** summary of the contribution ratios of capacitive capacity and diffusion-controlled capacity; **c, d** capacitive contribution analyzed through Trasatti's method, in which *q* and *v* are charge stored and scan rate, respectively. The maximum charge that can be stored in the anhydrous RuO_2 is only 126.1 C/g. Meanwhile, only 61.5% capacity is from the outer surface (77.6 C/g), which will lead to a modest rate capability of the anhydrous RuO_2.

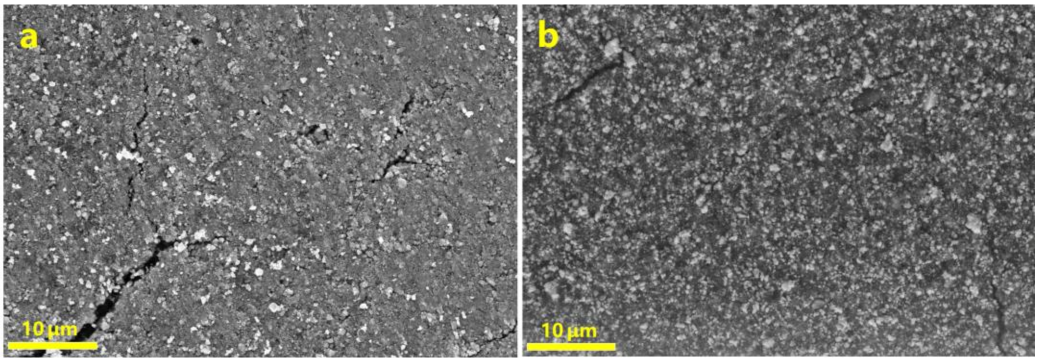

Fig. S13 SEM images of the $RuO_2{\cdot}H_2O$ cathode **a** before and **b** after 10000 charge/discharge cycles at 20 A/g

Supplementary References

[S1] Y. Jin, L. Zou, L. Liu, M.H. Engelhard, R.L. Patel et al., Joint charge storage for high - rate aqueous zinc - manganese dioxide batteries. Adv. Mater. **31**, 1900567 (2019). https://doi.org/10.1002/adma.201900567

[S2] H. Pan, Y. Shao, P. Yan, Y. Cheng, K.S. Han et al., Reversible aqueous zinc/manganese oxide energy storage from conversion reactions. Nat. Energy **1**, 16039 (2016). https://doi.org/10.1038/nenergy.2016.39

[S3] M. Chamoun, W.R. Brant, C.W. Tai, G. Karlsson, D. Noréus, Rechargeability of aqueous sulfate Zn/MnO_2 batteries enhanced by accessible Mn^{2+} ions. Energy Storage Mater. **15**, 351-360 (2018). https://doi.org/10.1016/j.ensm.2018.06.019

[S4] Y. Huang, J. Mou, W. Liu, X. Wang, L. Dong, F. Kang, C. Xu, Novel insights into energy storage mechanism of aqueous rechargeable Zn/MnO_2 batteries with participation of Mn^{2+}. Nano-Micro Lett. **11**, 49 (2019). https://doi.org/10.1007/s40820-019-0278-9
